# Visualizing County-Level Data to Target Dental Safety-Net Programs for Children

**DOI:** 10.5888/pcd18.200488

**Published:** 2021-03-11

**Authors:** Erin K. Hamilton, Jorge Bernal, Mei Lin, Gina Thornton-Evans, Susan O. Griffin

**Affiliations:** 1 CyberData Technologies, Inc, Herndon, Virginia; 2 Georgia Department of Public Health, Atlanta, Georgia; 3 Division of Oral Health, Centers for Disease Control and Prevention, Atlanta, Georgia

**Figure:**
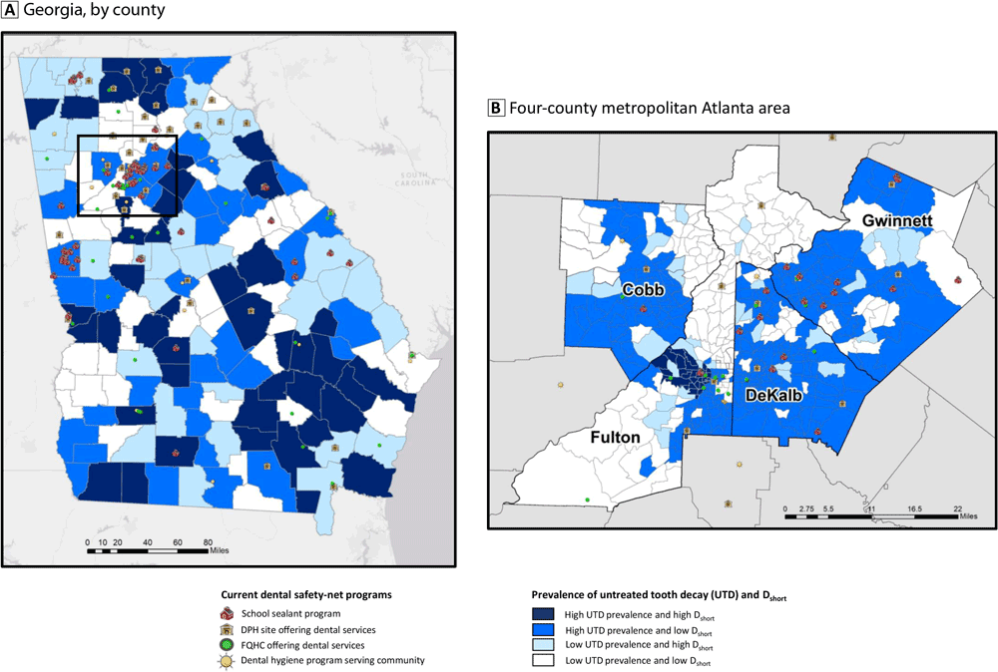
Current dental safety-net programs and areas of need for children aged 6 to 9 years. A, In Georgia, by county. Inset indicates the metropolitan Atlanta area. B, Metropolitan Atlanta area, by census tract. Maps were created by a data visualization tool that can be used to evaluate allocation of dental safety-net programs across the state and to inform decision makers on future resource needs and allocation. D_short_ represents the severity of a dental workforce shortage and is quantified as the number of full-time equivalent dental practitioners required to make the area a nonshortage area. Data sources: Lin et al (7), Health Resources and Services Administration (8), Georgia Department of Public Health (DPH) Oral Health Program (9), Georgia Primary Care Association (10), and Georgia Oral Health Coalition (11). Abbreviations: D_short_, shortage of dental practitioners; FQHC, federally qualified health center; UTD, untreated tooth decay.

## Background

More than 19% of third-graders in Georgia had untreated dental caries (tooth decay) in 2016–2017 (1). The national average among children of similar age (6–9 y) was 15.5% in 2013–2016 (2). Untreated tooth decay can cause pain and infection and impair eating, speaking, and learning. Among children it can lead to missed school days and lower academic performance (3). The most recent US data indicated that 34 million school hours were missed in 2007 as a result of acute unplanned dental care needs (4).

Dental sealants (5), topical fluoride (6), and restorative care are effective in preventing tooth decay. Most caries-prevention programs are implemented at the local level. The prevalence of untreated tooth decay, however, varies by geographic area — ranging from 8.2% to 32% among third-graders across 29 states during 2013–2016 (1). By county, modeled estimates among children aged 6 to 9 years nationwide ranged from 4.9% to 65.2% (7). Thus, having local data on the risk of untreated tooth decay and dental workforce capacity is critical to effectively target dental safety-net programs for children at highest risk of untreated tooth decay.

We developed a data visualization tool that maps county-level need for caries prevention and treatment programs and the distribution of dental safety-net programs in Georgia. This tool can be used by public health decision makers to 1) assess how well dental safety-net programs are currently allocated and 2) plan and target future programs.

## Data and Methods

We estimated the need for dental safety-net programs for each of the 159 Georgia counties and for each census tract in the 4 metropolitan Atlanta counties: Cobb (120 census tracts), DeKalb (143 census tracts), Fulton (202 census tracts), and Gwinnett (113 census tracts). Level of need was based on the estimated prevalence of untreated tooth decay and the severity of a dental workforce shortage (D_short_). We obtained data on the prevalence of untreated tooth decay from a study that estimated this information for children aged 6 to 9 years (7). That study generated county and census-tract estimates based on a multilevel regression and poststratification method applied to data on caries and sociodemographic characteristics from the National Health and Nutrition Examination Survey 2005–2010 linked with various area-level data at census tract, county, and state levels (7). We defined counties with an estimated prevalence of untreated tooth decay at or above the state’s median (20.1%) as having a high prevalence of untreated tooth decay (range, 20.1%–49.5%) and counties with a prevalence below the median as having a low prevalence of untreated tooth decay (range, 8.5%–19.9%).

We used data from the Health Resources and Services Administration (HRSA) (8) on the shortage of dental practitioners in each Georgia county designated as a geographic or population-based Dental Health Professional Shortage Area (DHPSA). For these counties, HRSA provides estimates of the number of full-time equivalent dental practitioners required to make the county a nonshortage area. We used the term “D_short_” to indicate levels of dental shortages; the higher the D_short_ values, the greater the shortage. Non-DHPSA counties are not assigned a value by HRSA, so we assigned a value of 0 to these counties. We used D_short_ instead of DHPSA designation as an indicator of workforce capacity because DHPSA designation was less specific — more than three-quarters of Georgia counties were DHPSAs. We designated counties and metropolitan Atlanta census tracts with values at or above the state’s median (1.34) as high D_short_ areas (range, 1.34–29.43) and census tracts below the median (range, 0–1.31) as low D_short_ areas.

We assigned counties to 1 of 4 categories: 1) low prevalence of untreated tooth decay and low D_short_, 2) low prevalence of untreated tooth decay and high D_short_, 3) high prevalence of untreated tooth decay and low D_short_, and 4) high prevalence of untreated tooth decay and high D_short_. We designated counties in category 4 as having the greatest need for dental safety-net programs and counties in category 1 as having the least need. We designated counties in category 3 as having a greater need for dental safety-net programs than those in category 2 because of their higher prevalence of untreated tooth decay.

We then overlaid information on current Georgia dental safety-net programs onto county need for such programs. Dental safety-net programs included 1) state-sponsored school sealant programs, 2) county-funded public health departments offering dental services, 3) federally qualified health centers offering dental services, and 4) dental hygiene programs providing community dental services. We obtained information on school sealant programs from the Georgia Department of Public Health Oral Health Program (9), information on federally qualified health centers from the Georgia Primary Care Association (10), and information on public health department dental sites and dental hygiene programs from the Georgia Oral Health Coalition (11). We generated maps by using ArcGIS version 10.5 and ArcGIS online (Esri).

## Highlights

Our visualization indicated good allocation of scarce dental public health resources. Of the 131 dental safety-net programs, 88 (67.2%) were in high-need counties, which is twice the number in low-need counties (43 or 32.8%). Many high-need counties, however, did not have dental safety-net programs. Among the 80 high-need counties, 52 had no programs (29 in category 4; 23 in category 3). In high-need areas, programs were more common in the metropolitan Atlanta area than in the rest of the state. This information is important for planning purposes, although the state may not be able to immediately address the problem of dental shortages in nonmetropolitan counties because of resource constraints and the higher cost (eg, longer driving time to transport dental professionals and portable sealant equipment) of serving areas farther away from the State Oral Health Program, which is based in Atlanta.

The maps also illustrate the importance of a granular visualization in areas with diverse populations, such as metropolitan areas. If only county levels are used, small pockets of need may be missed, as in Fulton County. Visualization at the census-tract level in Fulton County provides a better assessment of need and targeting.

## Action

Our mapping technique provides decision makers in Georgia with a visual tool for assessing how well current dental safety-net programs are allocated across the state and identify gaps in resource allocation where needs could be addressed in future program planning. The data used to generate these maps are publicly available for states nationwide and thus, these maps could be replicated throughout the United States.

## References

[1] Centers for Disease Control and Prevention. Oral health data. http://www.cdc.gov/oralhealthdata. Accessed June 4, 2020.

[2] US Department of Health and Human Services. Healthy people 2020: oral health. https://www.healthypeople.gov/2020/topics-objectives/topic/oral-health. Accessed June 4, 2020.

[3] Jackson SL , Vann WF Jr , Kotch JB , Pahel BT , Lee JY . Impact of poor oral health on children’s school attendance and performance. Am J Public Health 2011;101(10):1900–6. 10.2105/AJPH.2010.200915 21330579 PMC3222359

[4] Naavaal S , Kelekar U . School hours lost due to acute unplanned dental care. Health Behav Policy Rev 2018;5(2):66–73. 10.14485/HBPR.5.2.7

[5] Ahovuo-Saloranta A , Forss H , Hiiri A , Nordblad A , Mäkelä M . Pit and fissure sealants versus fluoride varnishes for preventing dental decay in the permanent teeth of children and adolescents. Cochrane Database Syst Rev 2016;(1):CD003067. 10.1002/14651858.CD003067.pub4 26780162 PMC7177291

[6] Marinho VC , Worthington HV , Walsh T , Clarkson JE . Fluoride varnishes for preventing dental caries in children and adolescents. Cochrane Database Syst Rev 2013;(7):CD002279. 10.1002/14651858.CD002279.pub2 23846772 PMC10758998

[7] Lin M , Zhang X , Holt JB , Robison V , Li C-H , Griffin SO . Multilevel model to estimate county-level untreated dental caries among US children aged 6-9years using the National Health and Nutrition Examination Survey. Prev Med 2018;111:291–8. 10.1016/j.ypmed.2017.11.015 29155223 PMC8029640

[8] Health Resources & Services Administration. HPSA find. https://data.hrsa.gov/tools/shortage-area/hpsa-find. Accessed March 19, 2020.

[9] Georgia Department of Public Health Oral Health Program. Programs and services. https://dph.georgia.gov/oral-health-services. Accessed March 16, 2020.

[10] Georgia Primary Care Association. Dental locations. https://georgiapca.org/places/dental. Accessed March 16, 2020.

[11] Georgia Oral Health Coalition. Access to care. https://www.gaohcoalition.org/resources/access-to-care. Accessed March 16, 2020.

